# Progressive postresection program (pPRP) after pancreatic resection: study protocol for a randomized controlled trial

**DOI:** 10.1186/s13063-016-1200-0

**Published:** 2016-02-10

**Authors:** Susanne Richter, Verena Uslar, Navid Tabriz, Thomas Mueser, Dirk Weyhe

**Affiliations:** Department of General and Visceral Surgery, Pius-Hospital Oldenburg, Oldenburg, Germany; Physiotherapy, Pius-Hospital Oldenburg, Oldenburg, Germany

**Keywords:** Intensified physiotherapy, Quality of life after pancreatic resection, Pancreatic cancer, SF-36 Health Survey, SF-8 Health Survey, EORTC QLQ-C30, EORTC QLQ-PAN26, Progressive postresection program, Follow-up pancreatic cancer, Walking program

## Abstract

**Background:**

At the time of initial diagnosis, only 15–20 % of patients with pancreatic cancer present with a resectable disease. Patients with pancreatic cancer face a poor prognosis. Progression-free survival and overall survival rates are very limited, so it is important to develop concepts to improve the quality of life for their remaining lives.

**Methods/design:**

The proposed trial is a randomized controlled intervention study. After pancreatic resection, the intervention group (cohort A, *n* = 30 patients) will take part in an intensified physiotherapy program consisting of endurance and muscle force exercises. The control group (cohort B, *n* = 30 patients) will take part in standard physiotherapy. Both groups will receive dietary counseling and, if necessary, substitution for endocrine/exocrine pancreatic insufficiency. Quality of life will be evaluated using the Short Form-8 Health Survey and the European Organization for Research and Treatment of Cancer QLQ-C30/QLQ-PAN26 questionnaires.

**Discussion:**

The aim of this study is to investigate whether intensive physiotherapy improves the quality of life of patients after pancreatic resection. If the results for the intervention group are positive, a multicenter study should be performed with appropriate statistical power. The progressive postresection program includes a structured follow-up after pancreatic resection. In this study, all patients will undergo abdominal computed tomography for follow-up 6 and 12 months postoperatively.

**Trial registration:**

German Clinical Trials Register DRKS00006786. Date of registration 1 October 2014.

## Background

In 2010, more than 16,000 people were diagnosed with pancreatic cancer in Germany, and almost as many died of this disease. The mean age of diagnosis is 71 years for men and 75 years for women. The age-standardized incidence and mortality rates are almost constant, but the absolute number of cases is increasing as a result of the aging population [1]. At the time of initial diagnosis, only 15–20 % of patients with pancreatic cancer present with a resectable disease [2]. Despite the improved treatment options, the relative 5-year survival rate has improved only from 2 % in 1975 to 8 % in 2010 [1, 3].

The clinical and scientific interest in the quality of life (QOL) of patients with cancer has been increasing since the 1990s [4]. Intensified physical training has been shown to measurably increase QOL and reduce fatigue levels of patients with cancer [5, 6]. So far, there are only a few studies that have dealt with the issue of QOL and developed concepts to improve the QOL of patients after pancreatic resection. Yeo et al. found that patients with pancreatic and periampullary cancer who have undergone resection benefit from a structured home-walking program with respect to fatigue, pain, physical functioning, and mental health. They modified the Every Step Counts graduated walking program to suit patients with pancreatic and periampullary cancer who underwent resection [5].

We have modified the walking program used by Yeo et al. with respect to length. Our program is designed to last 12 months after surgery instead of 3 months. Moreover, we combine the walking program with muscle force exercises, as we believe that the patients will benefit from a combination of endurance and muscle force exercises.

Health-related QOL is often assessed using the Short Form-36 Health Survey (SF-36). In this questionnaire, eight columns are used to assess QOL: vitality, physical functioning, bodily pain, general health perceptions, physical role functioning, emotional role functioning, social functioning, and psychological well-being [7].

The Short Form-8 Health Survey (SF-8) was developed as a shorter form of the SF-36. It was used in the Telephone Health Survey 2003, and it provides results that are comparable with those of the SF-36, the most widely used instrument internationally. Every item of the SF-8 presents one of the above-mentioned eight columns of the SF-36 questionnaire. The SF-8 questionnaire has two domains: the physical component score and the mental component score [8].

To specifically assess the QOL of patients with pancreatic cancer, the European Organization for Research and Treatment of Cancer (EORTC) developed and validated the QLQ-PAN26 questionnaire, which contains the domains “disease specific symptoms,” “functional status,” and “psychosocial components.” This is to be used in conjunction with the QLQ-C30 questionnaire. The QLQ-C30 questionnaire was also developed by the EORTC. It was validated to determine the QOL of patients with cancer [4]. Both sheets are available in validated German translations.

High-quality physical therapy and a motivating workout schedule could be key parameters for increasing the QOL of patients with pancreatic cancer. Thus, the main aim of this study is to investigate the effect of a new workout scheme on QOL and to ascertain the general feasibility of the planned approach (i.e., patient’s compliance with the workout schedule). In addition, a more outlined definition of clinically relevant improvement will be identified as part of this trial. The patient’s compliance might have an especially strong impact on the outcome of any later studies. Thus, it is important to ensure that the chosen schedule does not overstrain the patients and instead increases their QOL and overall motivation.

## Methods/design

The study protocol has been approved by the Commission for Impact Assessment Research and Ethics, Carl von Ossietzky University, Oldenburg, Germany (Drs. 59/2014).

### Study population

The study population will consist of patients scheduled for pancreatic resection for resectable pancreatic cancer, distal bile duct carcinoma, neuroendocrine tumor, periampullary carcinoma, duodenal carcinoma, and intraductal papillary mucinous neoplasm. Further inclusion criteria are age (≥18 years) and surgery and initial postoperative hospitalization at the Department of General and Visceral Surgery, Pius-Hospital, Oldenburg, Germany.

Exclusion criteria are lack of written consent, physical inability to participate in the intensified physiotherapy program, illiteracy, inability to communicate in the German language, physical or mental disability that precludes participation in the intensified physiotherapy program, and lack of compliance.

Individual criteria for discontinuation include withdrawal of the patient’s consent, a new cardiac risk profile in the form of myocardial infarction or congestive heart failure, complications after pancreatic resection in the form of postoperative mechanical ventilation past the first postoperative week, and physical or mental inability to participate in the intensified physiotherapy program.

### Study objectives

The primary aim of this study is the assessment of QOL in the intervention group after intensified physiotherapy 12 months postoperatively, using the SF-8 and the disease-specific EORTC QLQ-C30/QLQ-PAN26 questionnaires in the validated German translations. The longitudinal development of QOL will be measured using the SF-8 and the QLQ-C30/PAN26 questionnaires. The different domains of the questionnaires will be evaluated separately.

The secondary aims of this study include assessment of study dropouts, assessment of physical performance using the Short Physical Performance Battery and ergometry, and assessment of nutritional status using metabolic blood levels and the seven-site skinfold method. If possible, influence on 1-year survival rate, effects of potential adjuvant therapy on QOL, and tumor recurrence rate after 6 and 12 months will be investigated as well. This depends on the actual number of patients and the heterogeneity of the two study groups.

### Design and trial flow

**Fig. 1:**
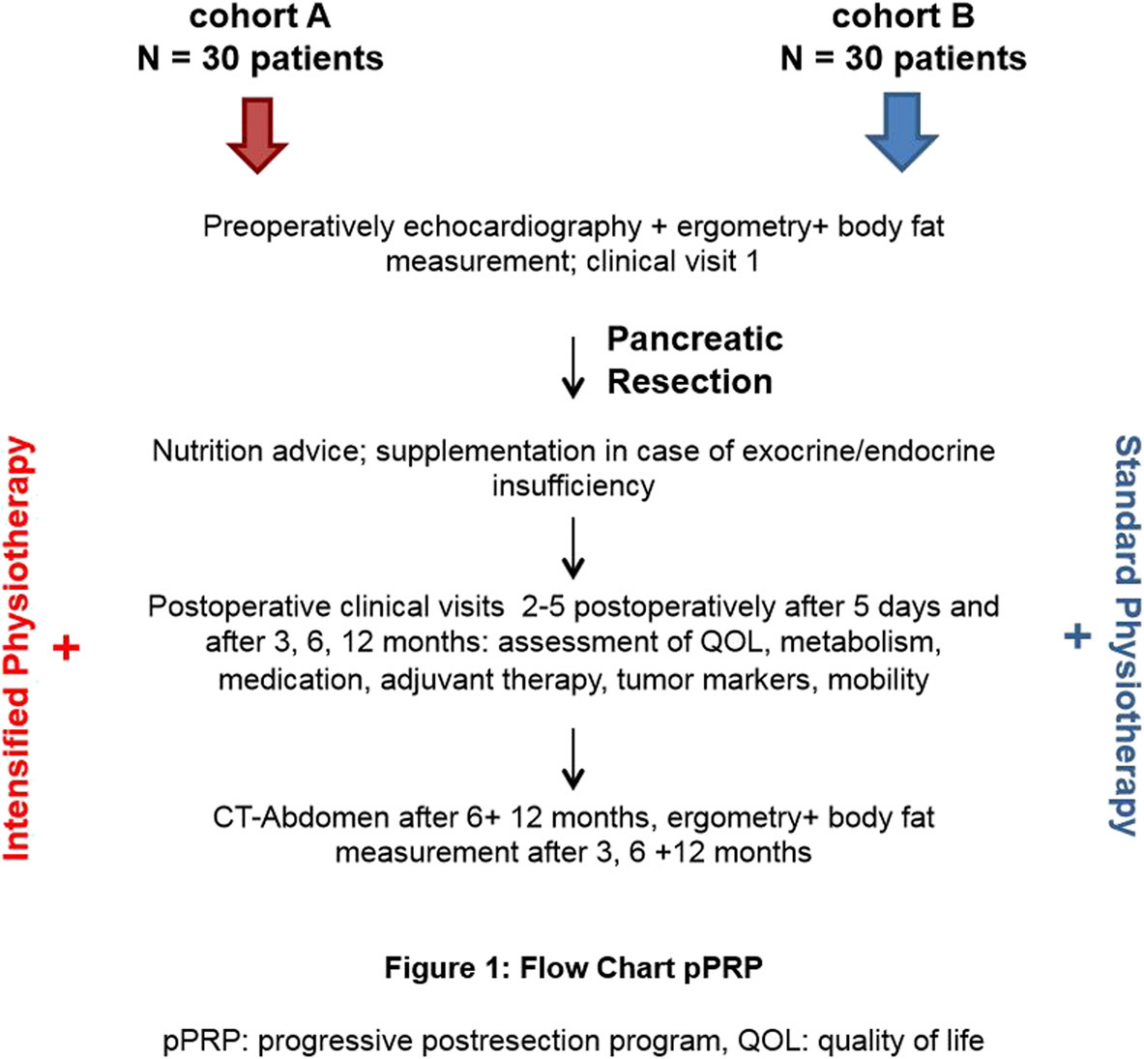
Flowchart for the progressive postresection program. *CT* computed tomography, *QOL* quality of life, *pPRP* progressive postresection program

Figure 1 shows the flowchart of this study.

For participation in this prospective randomized controlled intervention study, informed written consent is mandatory. Should the study yield positive results, a multicenter study with appropriate statistical power will follow.

The recruitment period is 24 months, and 60 patients are planned to be enrolled in the study. The intervention group (cohort A, *n* = 30 patients) will receive an intensified physiotherapy, whereas the control group (cohort B, *n* = 30 patients) will receive physiotherapy that is the standard treatment at the Pancreatic Cancer Center, Pius-Hospital, Oldenburg, Germany.

The follow-up of the patients enrolled in this study will be 12 months postoperatively. Evaluation of the data will take up to 6 months.

The standard physiotherapy of the control group (cohort B) is individualized on the basis of the physical condition of the patient and is scheduled for 20 minutes on 5 days per week. It consists of individual therapy with relaxation and mobilization exercises, walking, and possibly even climbing stairs. It is limited to the duration of the hospital stay.

The intensified physiotherapy of the intervention group (cohort A) will follow the scheme described below:

1. *From the first 24 h after extubation*: Ten minutes of activity three times per day with the aid of a bed bicycle; target of 50–70 % of maximal heart rate within the first postoperative week.
2. *From the second postoperative week*: Fifteen minutes of walking three times per day plus muscle training using a TheraBand resistance band and 2-kg dumbbells and modified squats 5 days per week.
3. *After discharge*: Every Step Counts graduated walking program plus continuation of muscle exercises three times per week.

The physiotherapy program will start within the first 24 h after extubation with three rounds of bed cycling, each round taking 10 minutes. Starting from the second postoperative week, patients are supposed to walk three times per day (15 minutes each) and do muscle exercises on 5 days per week. Upon discharge from the rehabilitation clinic, up until the 12th postoperative month, patients are instructed to follow the Every Step Counts graduated walking program with the minor modifications for resected pancreatic and periampullary cancer patients made by Yeo et al. [5] (Table 1), and they will be asked to continue their muscle exercises three times per week.

**Table 1:** Graduated walking program

Time point	Exercise program
Month 1 after discharge	Warm-up: Walk slowly for 5 minutes
	Brisk walking: 10 minutes
	Cool-down: Walk slowly for 5 minutes
Month 2 after discharge	Warm-up: Walk slowly for 5 minutes
	Brisk walking: 20 minutes
	Cool-down: Walk slowly for 5 minutes
From month 3 after discharge	Warm-up: Walk slowly for 5 minutes
	Brisk walking: 25–30 minutes
	Cool-down: Walk slowly for 5 minutes

From Yeo et al. [5]

To minimize further rehabilitation therapy as a confounding factor, almost all of our patients will receive rehabilitation therapy in the same clinic. After discharge, implementation of the enhanced physiotherapy will be controlled monthly by study nurses via telephone. The patients will be asked the following questions:

1. Do you perform the intensified physiotherapy regularly?
2. If not, for what reason?

Preoperatively, all patients will undergo transthoracic echocardiography (TTE), as well as an ergometric test to check for a potential cardiac risk profile. In addition, a body fat measurement will be performed using the seven-site skinfold method. For this purpose, the thoracic, abdominal, leg, hip, armpit, triceps, and back pleat measurements are done with a caliper. The fat-free and fatty tissue weights, as well as the body fat percentage, can be calculated with this method and are recorded with the patient’s age and body weight (Table 2).

**Table 2:** Evaluation of body fat in adults according to the World Health Organization criteria

Age in years	Low	Healthy	Increase	Obese
Body fat rating female (% fat)				
20–39	<21	21–33	33–39	>39
40–59	<23	23–34	34–40	>40
>60	<24	24–36	35–42	>42
Body fat rating male (% fat)				
20–39	<8	8–22	20–25	>25
40–59	<11	11–22	22–28	>28
>60	<13	13–25	25–30	>30

The scheduled clinical visits take place 2 days preoperatively as well as 5 days and 3, 6, and 12 months postoperatively with the following measurements:

- 2 days preoperatively (+ TTE + ergometry + body fat measurement)
- 5 days postoperatively
- 3 months postoperatively (+ ergometry + body fat measurement)
- 6 months postoperatively (+ CT of the abdomen + ergometry + body fat measurement)
- 12 months postoperatively (+ CT of the abdomen + ergometry + body fat measurement)

The following will constitute the content of the clinical visits:

1. Assessment of QOL: SF-8, EORTC QLQ-C30/QLQ-PAN26
2. Documentation of medication/adjuvant treatment (radiotherapy, chemotherapy)
3. Documentation of the tumor markers CEA and CA 19-9
4. Assessment of mobility: Short Physical Performance Battery
5. After 6 and 12 months: CT of the abdomen
6. Preoperatively and at 3, 6, and 12 months postoperatively: ergometry + body fat measurement
7. Metabolism diagnostics

The metabolism diagnostic measurements will include size, weight, and body mass index (BMI). The laboratory parameters measured will include albumin, iron, transferrin, ferritin, vitamin B_12_, homocysteine, folic acid, magnesium, zinc, calcium, parathyroid hormone, vitamin D, hemoglobin, glycated hemoglobin A1c (HbA1c), cholesterol, high-density lipoprotein (HDL) cholesterol, low-density lipoprotein (LDL) cholesterol, and triglycerides.

Postoperatively, the patients will receive dietary counseling during the course of their hospital stay. For patients with endocrine pancreatic insufficiency, pancreatic enzyme substitution and insulin levels are optimized and diabetes education is provided. All patients are offered psycho-oncological care.

### Sample size considerations

The study is designed as a pilot study. Therefore, no sample size calculation was performed. The sample size of 60 patients given here is derived from the feasibility of patient recruitment within 2 years.

### Documentation and data handling

Protocol-required information will be documented in the case report form, and it will be reviewed and signed by the investigator or by a designated subinvestigator. Data transfer will be carried out pseudonymously.

### Randomization and blinding

Allocation into the two study groups will be completely randomized using the program Randomizer.at. The study cannot be blinded, as patients of the intervention group will receive a different physiotherapy program and need to have a bed bicycle.

### Statistical methods

Data analysis will include intention-to-treat and per-protocol analyses. The QOL of patients evaluated with the SF-8 and EORTC QLQ-C3/QLQ-PAN26 questionnaires will be illustrated in histograms. We will evaluate the QOL using the following schedule: 2 days preoperatively and 5 days and 3, 6, and 12 months postoperatively. Furthermore, the course of QOL will be evaluated with a longitudinal regression model.

A multiple linear regression model that includes the following covariates will be used: type of surgery (distal pancreatectomy, pancreaticoduodenectomy, total pancreatectomy), cancer (yes or no), age, BMI, mobility, type of physiotherapy (intensified or standard). We will also perform a covariate analysis.

### Ethical issues

The trial will be performed according to the Declaration of Helsinki. The study protocol was approved by the Commission for Impact Assessment Research and Ethics, Carl von Ossietzky University, Oldenburg, Germany (Drs. 59/2014). Informed consent will be obtained and validated using both the physician’s and patient’s signatures. Participation is voluntary, and patients can quit the trial at any time without disclosure of their motives for withdrawal and without fear of subsequently receiving poor medical care. In the case of withdrawal, relevant data will be deleted if desired by the patient. Patient names and all other confidential information are subject to medical confidentiality under the German Data Protection Act. Transmission of data will be done in an encrypted format. Others not involved in the trial will have no access to original documents.

## Discussion

The absolute number of patients with pancreatic cancer is increasing, and patients with pancreatic cancer generally have a poor prognosis, so the QOL of patients with pancreatic cancer is of clinical and scientific interest. The complexity of the molecular biology underlying pancreatic cancer makes a breakthrough in the treatment options in the near future highly unlikely. For this reason, and because the rates of traditional outcome parameters such as progression-free survival and overall survival are low, it is very important to develop concepts to improve the QOL of patients with pancreatic cancer in their remaining lives [10]. There are indications that an improved QOL is associated with an improved overall survival rate [10].

As mentioned above, intensified physical training has been shown in particular to measurably increase QOL and reduce the fatigue level of patients with cancer [5, 6]. There are only a few studies that have dealt with the issue of QOL and developing concepts to improve the QOL of patients after pancreatic resection. Yeo et al. described a benefit for patients with resected pancreatic and periampullary cancer from taking part in a home walking program based on the Every Step Counts graduated walking program [5]. They found significant improvements in fatigue levels, physical functioning, and health-related QOL [5]. We believe that our patients will profit from muscle force exercises in addition to endurance exercises. For this reason, we combine a modified version of the Every Step Counts graduated walking program with muscle force exercises. Furthermore, we use the general questionnaire SF-8 as well as the disease-specific questionnaires EORTC QLQ-C30 and QLQ-PAN26 to evaluate the QOL of our patients.

The aim of this study is to investigate whether intensive physiotherapy, consisting of a combination of endurance and muscle force exercises, improves the QOL of patients after pancreatic resection for pancreatic cancer, distal bile duct carcinoma, neuroendocrine tumor, periampullary carcinoma, duodenal carcinoma, or intraductal papillary mucinous neoplasm.

Our hypothesis is that, with the exception of tumor recurrence, QOL should increase more for the intervention group than for the control group, regardless of the reason for surgery, if the type of physical therapy really has an impact on QOL. To be considered clinically relevant, this should be visible even in the heterogeneous study groups employed here.

The main objective criterion is a potential improvement in QOL in the intervention group after intensified physiotherapy 12 months postoperatively as measured by the SF-8 and the EORTC QLQ-C30/QLQ-PAN-26 questionnaires. This pilot study should help us to find an outlined definition of improvement, which may then be implemented in a larger (multicenter) study with appropriate statistical power.

### Trial status

Enrollment of the first patient is planned for February 2016.

## Abbreviations

BMI: body mass index
CA 19-9: cancer antigen 19-9
CEA: carcinoembryonic antigen
CT: computed tomography
EORTC: European Organization for Research and Treatment of Cancer
Hb: hemoglobin
HbA1c: glycated hemoglobin A1c
HDL: high-density lipoprotein
LDL: low-density lipoprotein
QOL: quality of life
SF-8: Short Form-8 Health Survey
SF-36: Short Form-36 Health Survey
TTE: transthoracic echocardiography
